# Supplementary material for: A Method for Evaluating Robustness of Limited Sampling Strategies—Exemplified by Serum Iohexol Clearance for Determination of Measured Glomerular Filtration Rate
Source: Pharmaceutics. 2023 Mar 27;15(4):1073. doi: 10.3390/pharmaceutics15041073 (PMC10143161; doi:10.3390/pharmaceutics15041073)
Supplement: Supplementary file 1 [file pharmaceutics-15-01073-s001.zip › pharmaceutics-2227146-supplementary.pdf]

SUPPLEMENTARY MATERIAL

Population pharmacokinetic model

A covariate free version of the original model [9] was developed, as the present method for simulation does not allow for covariates. The model achieved a mean prediction error -0.28 mg/mL and a relative root mean squared error of 3.7%.

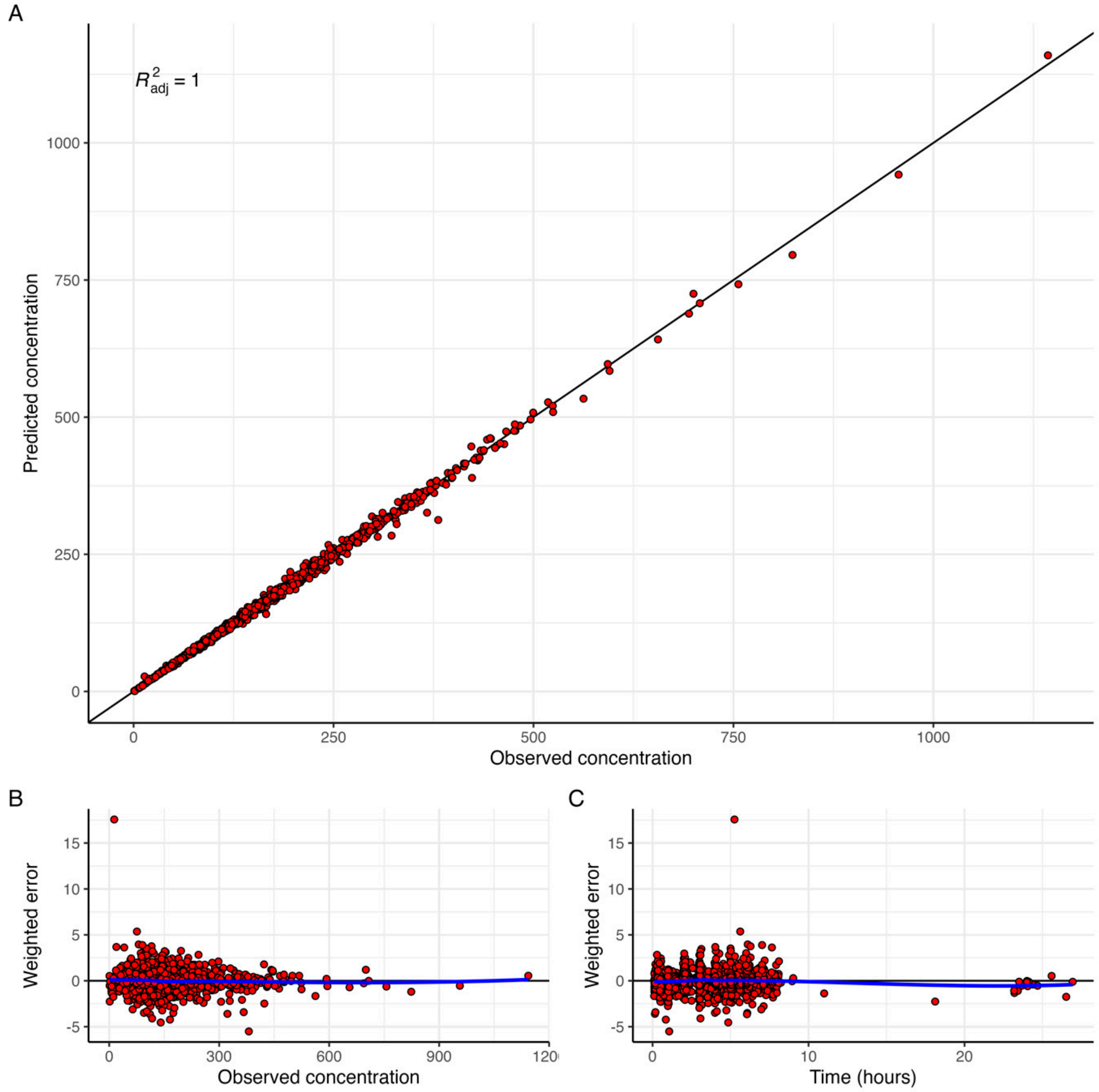

Figure S1. Population pharmacokinetic model performance plots for the covariate-free model, including A) observed-predicted plot, B) weighted error across observed concentrations, and C) weighted error across sample time. Solid black lines represent the unity line, and the solid blue lines in B) and C) indicate the loess line.

Supplementary Table 1. Population pharmacokinetic performance metrics for the covariate-free model.

Metric	Value
Mean prediction error	-0.28
Mean weighted prediction error	0.03
Mean squared prediction error	30.67
Root-mean squared error (RMSE)	5.54
% RMSE	3.66
Mean weighted squared prediction error	1.49
Bias-adjusted mean squared prediction error	30.59
Bias-adjusted mean weighted squared prediction error	1.49

Supplementary Code S1

Implementation of the population pharmacokinetic model in mrgsolve for R.

```
$PROB

Population pharmacokinetic model of iohexol

$SET

delta = 0.1

end = 24

$PLUGIN Rcpp mrgx

$PARAM @annotated

TVCL : 2.89 : Typical value of clearance

TVV1 : 10.36 : Volume of central compartment

TVV2 : 9.2 : Volume of peripheral compartment

TVQ : 10.65 : Inter-compartment bloodflow

$MAIN

double WT = TVWT;

double CL = TVCL;

double V1 = TVV1;

double V2 = TVV2;

double Q = TVQ;

double KCP = Q / V1;

double KPC = Q / V2;

$PKMODEL

ncmt=2

depot = FALSE

$CMT @annotated

CENT : Central compartment

PERI : Peripheral compartment

$TABLE

capture CP = CENT/V1;

capture CLi = CL;

$SIGMA @annotated

ADD : 0 : Additive residual

PROP: 0 : Proportional residual error
```
